# Development of a duplex qPCR for the differentiation of a live attenuated *Escherichia coli* *aroA* mutant vaccine strain from field isolates in chickens

**DOI:** 10.1371/journal.pone.0278949

**Published:** 2022-12-19

**Authors:** Kirsten Leurs, Evy Goossens, Henrik Christensen, Jacques G. Mainil, Dieter Vancraeynest, Richard Ducatelle, Filip Van Immerseel

**Affiliations:** 1 Department of Pathobiology, Livestock Gut Health Team (LiGHT) Ghent, Pharmacology and Zoological Medicine, Faculty of Veterinary Medicine, Ghent University, Merelbeke, Belgium; 2 Department of Veterinary Microbiology, Royal Veterinary and Agricultural University, Frederiksberg C, Denmark; 3 Bacteriology Department of Infectious Diseases, Faculty of Veterinary Medicine, Centre for Fundamental and Applied Research in Animals and Health (FARAH), University of Liège (ULiège), Liège, Belgium; 4 Zoetis, Parsippany, New Jersey, United States of America; University of Illinois College of Medicine, UNITED STATES

## Abstract

Avian pathogenic *Escherichia coli* (APEC) can cause colibacillosis in poultry, characterised by localised or systemic infections. Colibacillosis is considered one of the leading causes of economic losses in the poultry industry due to reduced performance, increased mortality, treatment costs and carcass condemnations. A live attenuated *Escherichia coli* O78 *aroA* gene mutant is widely used to prevent disease. However, no effective strategies to differentiate the vaccine strain from field strains are available, hampering follow-up of vaccination campaigns. In the current study, we report a PCR-based method to simultaneously detect the vaccine strain by targeting the vaccine-specific mutation in the *aroA* gene, as well as the wild type *E*. *coli* strains by targeting the *xanQ* gene. The specificity of this PCR was evaluated using 123 *E*. *coli* isolates, form which 5 WT *aroA* auxotrophic strains (WT strains with a natural *aroA* deficiency), as well as 7 non-*Escherichia* isolates. The PCR showed 100% sensitivity of the *xanQ* primers for *E*. *coli* detection and 100% sensitivity of the Δ*aroA* primers for the vaccine strain. In order to allow quantification of the vaccine strain in complex samples containing many different *E*. *coli* strains and other related organisms, such as chicken faeces, a probe-based duplex qPCR was developed. The limit of detection (LOD) of this duplex qPCR method was 8.4*10^3^ copies/g faeces. The specificity of the duplex qPCR was confirmed by determining both the vaccine strain levels, and the total *E*. *coli* load in intestinal digesta from both vaccinated and non-vaccinated birds. *E*. *coli* could be detected in both vaccinated and non-vaccinated birds. The duplex qPCR was specific for the vaccine strain as this strain was detected in all vaccinated birds, whereas no signal was detected in non-vaccinated birds. The duplex qPCR is helpful in monitoring colonization and shedding of the vaccine strain.

## Introduction

*Escherichia (E*.*) coli* is commonly present in the gastrointestinal tract of vertebrates. While most *E*. *coli* strains are benign, some are virulent and capable of inducing disease. Avian pathogenic *E*. *coli* (APEC) strains are the causative agents of colibacillosis, an economically important disease in domestic poultry [1]. The most common form of the disease begins either as a respiratory tract infection (causing airsacculitis and pneumonia) or salpingitis combined with peritonitis. When the bacteria reach the bloodstream and spread systemically, this can lead to colisepticaemia, characterized by fibrinous lesions in different organs (pericarditis, peritonitis, perihepatitis, salpingitis, cellulitis and splenitis) [2–6]. In the past, control of severe colibacillosis outbreaks has relied heavily on antibiotic treatments. This approach, however, is now under pressure because of major issues of antimicrobial resistance in *E*. *coli* [7]. Therefore, vaccination has become a common strategy to control APEC infection in commercial poultry. One such vaccine commonly used in the field is Poulvac® *E*. *coli* (Zoetis, Parsippany, New York, USA), a live attenuated strain of *E*. *coli* derived from a virulent O78:K80 APEC strain by mutating the *aroA* gene through allelic exchange. This defined *aroA* deletion mutant is deficient in the biosynthesis of aromatic acids and therefore cannot survive in the environment [8–10].

Differentiation of vaccine strains from field strains is recommended by various international organizations involved in the control of animal diseases such as the World Organization of Animal Health (WOAH) and the Food and Agriculture Organization of the United Nations (FAO). It is even a legal obligation for live *Salmonella* vaccines intended for vaccination of laying hens in the EU (regulation 1177/2006/EC). This is not yet the case for *E*. *coli*, but considering the ubiquitous presence of *E*. *coli* in the intestinal tract of animals, a similar strategy is highly recommendable. Identifying a specific bacterial strain can be problematic, mainly when derived from complex samples such as intestinal content. For example, the classical selective and indicative culture media usually allow selective growth and identification up to the family or genus level. Detection and identification of a single bacterial strain in a complex mixture typically rely on a PCR-based technology. To date, the differentiation of the commercial *aroA* mutant vaccine strain from other *E*. *coli* strains is achieved either via (i) the absence of growth on minimal agar whilst presenting growth on MacConkey agar [5, 11]; (ii) discrimination based on pulsed-field gel electrophoresis (PFGE) band patterns [11]; or (iii) a PCR developed by La Ragione *et al*. (2013) [10], in which the amplicon length between vaccine or field strain DNA shows only a marginal difference in amplicon length, which complicates the interpretation of the result.

Therefore, the current study aimed to develop a more robust PCR approach to discriminate between the *aroA* mutant and field strains. In addition, the primers designed in this study were used to establish a duplex quantitative PCR (qPCR) to simultaneously quantify *aroA* mutant and total *E*. *coli* levels in the intestinal tract of poultry, a tool which can be useful in the follow-up of vaccination campaigns.

## Material and methods

### Bacterial strains and culture conditions and genomic DNA extraction

A live attenuated *aroA* mutant vaccine strain, derived from a virulent O78:K80 APEC strain, hereafter referred to as Δ*aroA*, was used throughout this study (Poulvac® *E*. *coli*, Zoetis, Charles City Manufacturing site, USA). In addition, 132 *E*. *coli* strains including 5 aromatic amino acid auxotrophic strains (*E*. *coli* strains isolated from chickens that harbor a WT *aroA* gene, but were found to be unable to synthesize aromatic amino acids and therefore are phenotypically indistinguishable from the Δ*aroA* vaccine strain using standard plating methods [11]), as well as seven non-*Escherichia* strains were used to optimise and validate the PCR (S1 Table). All strains were routinely grown according to their growth preferences (aerobic or anaerobic) at 37°C. *E*. *coli* strains were grown on MacConkey agar, *Clostridium perfringens* on Columbia blood agar, *Salmonella* strains on Brilliant Green agar, *Bacillus* on Luria-Bertani agar and the *Lactobacillus* strain on De Man, Rogosa and Sharpe (MRS) agar. Genomic DNA was isolated from all strains using the alkaline lysis method by resuspending a single bacterial colony (or the pellet from 100μl culture) in 20 μl lysis buffer (0.25% SDS, 50 mM NaOH). After boiling at 95°C for 5 min, 180 μl of nuclease-free water was added and centrifuged for 5 min at maximum speed. The DNA was stored at -20°C until further use.

The analytical sensitivity of the developed duplex qPCR was assessed by comparing the Δ*aroA* count with the qPCR results. Therefore, the Δ*aroA* strain was grown overnight in Luria-Bertani Broth (LB) at 37°C with gentle shaking. A 10-fold dilution was made from this overnight culture and titrated on LB agar plates and colony-forming units (CFU)/ ml were counted the day after. From this 10-fold dilution, 100 μl culture was pelleted and the pellet was used to isolate genomic DNA using alkaline lysis as described above.

### Target selection, primer and probe design

To develop a PCR that can discriminate between wild type (WT) *E*. *coli* and Δ*aroA*, both the wild type *aroA* gene (GenBank accession number CP004009.1) and the *aroA* mutant gene as created by La Ragione *et al*. (2013) were targeted [10]. To develop a duplex PCR to simultaneously determine the amount of Δ*aroA* relative to the total amount of *E*. *coli* (both WT and Δ*aroA*), the *xanQ* gene was targeted in addition to the *aroA* gene. The usability of this *xanQ* gene for the reliable detection of *E*. *coli* was previously described [12].

**Fig 1:**
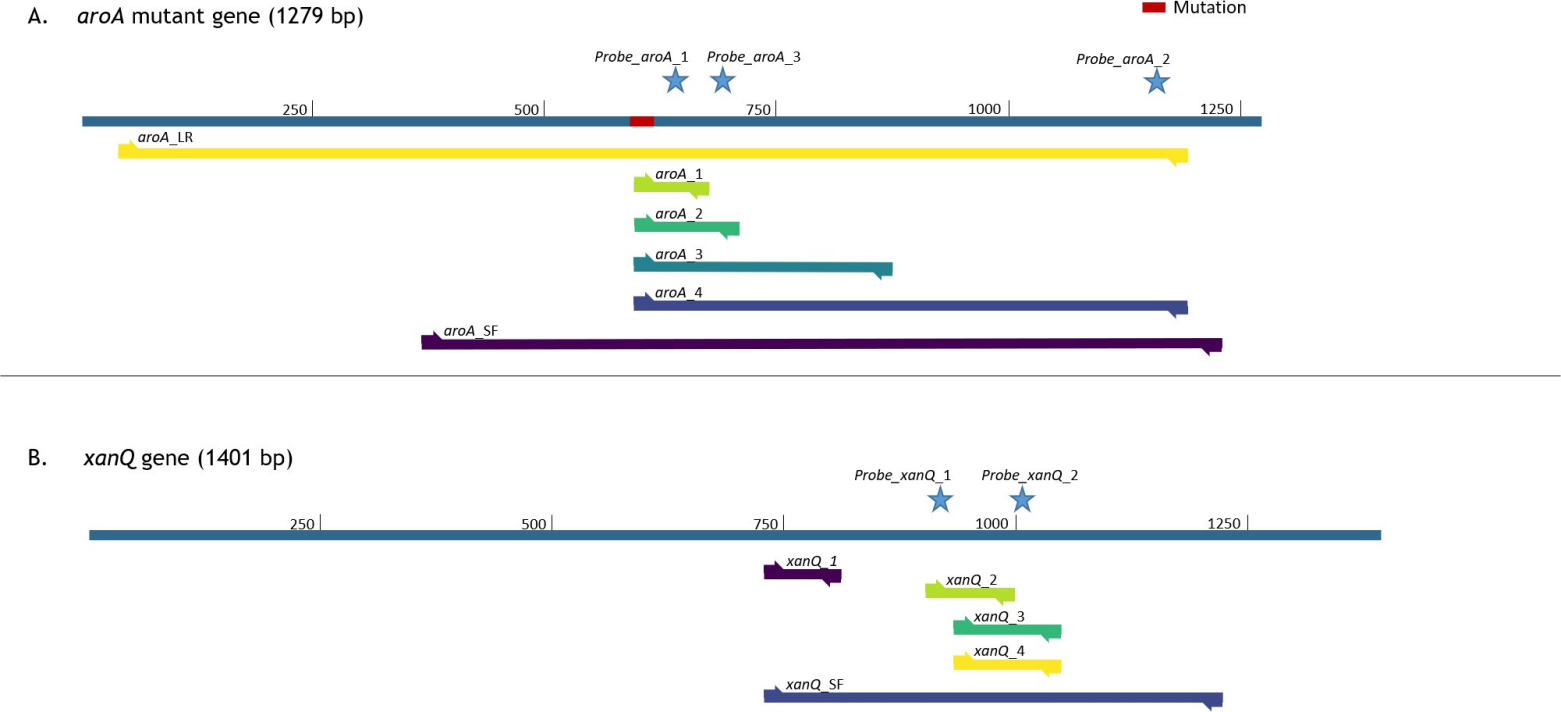
Graphical representation of the primers and probes used in this study. (A) The *aroA* mutant gene (1279 bp) is schematically represented by the blue line on which the mutation is indicated in red. Underneath this line, the amplicons generated by the different primer pairs (arrows) are represented. Above the schematic gene representation, the location of the probes is indicated with a star. Since this figure depicts the *aroA* mutant gene, the primer pair *aroA*_WT cannot be represented. (B) A similar representation for the *xanQ* gene (1401 bp) in blue with probes (above) and primers (below).

Primers and primer/probe sets were designed using the PrimerQuest^TM^ software (IDT, Integrated DNA Technologies Inc., Coralville, IA, USA). The specificity of the designed primers and probes towards *E*. *coli* was confirmed using Primer Blast analysis [13]. 6-carboxyfluorescein (FAM) and hexachlorofluorescein (HEX) conjugated to the 5’ ends of the probes were used as fluorescent reporter dyes to detect amplification products specific for *aroA* (FAM) or *xanQ* (HEX). Because of its high fluorescent signal, FAM is known to be a suitable dye to detect low-copy transcripts. As *E*. *coli* is a commensal, the total amount of *E*. *coli* (both WT and Δ*aroA*) in intestinal content samples will exceed the amount of Δ*aroA*. Therefore, it was opted to couple a FAM signal to the probe for the *aroA* mutation (minority part) and a HEX signal to detect the *xanQ* gene (majority part). To obtain optimal signal detection, with minimal background fluorescence, all probes were designed as double-quenched probes, using a 3’ Iowa Black®FQ and ZEN quencher. The ZEN quencher has been reported to increase the signal sensitivity by decreasing the background fluorescence. The Iowa Black® FQ quencher was selected because it has a broad absorbance spectrum ranging from 420 to 620 nm with a peak absorbance at 531 nm and therefore overlaps with the emission spectra of FAM (excitation wavelength of 495 nm and emission wavelength of 520 nm) and HEX (excitation wavelength of 538 nm and emission wavelength of 555 nm). All the different primers and probes developed in this study were synthesized by IDT (Coralville, IA, USA) and are listed in Tables 1 and 2. Fig 1 is a graphical representation of these primers and probes on the genes.

**Table 1:** List of primer sequences.

**Primer pair**	**Primer name**	**Sequence**	**Target gene**	**Amplicon (bp)**		**Analysis**	**Reference**
				**WT**	Δ*aroA*		
*aroA*_LR	*aroA*_LR_fw	5’-ATCCCTGACGTTACAACC-3’	*aroA*	1236	1161	PCR	[10]
	*aroA*_LR_rev	5’-TCCGCGCCAGCTGCTCGA-3’					[10]
*aroA*_1	*aroA*_fw	5’-AGCCCGGGCTAAAGATCTTA-3’	Δ*aroA*	-	82	PCR / qPCR	This study
	*aroA*_1_rev	5’-GAAAGTAAGAAGCCGAAGATGC-3’					This study
*aroA*_2	*aroA*_fw	5’-AGCCCGGGCTAAAGATCTTA-3’	Δ*aroA*	-	114	PCR / qPCR	This study
	*aroA*_2_rev	5’-TACAGTGCCGCCTTTGATTG-3’					This study
*aroA*_3	*aroA*_fw	5’-AGCCCGGGCTAAAGATCTTA-3’	Δ*aroA*	-	281	PCR / qPCR	This study
	*aroA*_3_rev	5’-ATCGCCGCATCAGGAATATG-3’					This study
*aroA*_4	*aroA*_fw	5’-AGCCCGGGCTAAAGATCTTA-3’	Δ*aroA*	-	601	PCR / qPCR	This study
	*aroA*_LR_rev	5’-TCCGCGCCAGCTGCTCGA-3’					[10]
*aroA*_WT	*aroA*_WT_fw	5’-TCACACTCAATCTGATGAAGACGTTTG-3’	*aroA*	660	-	PCR	This study
	*aroA*_LR_rev	5’-TCCGCGCCAGCTGCTCGA-3’					[ 10 ]
*aroA*_SF	*aroA*_SF_fw	5’-CCTGGGCGGGGCGAAGAT-3’	*aroA*	944	869	Standard fragment PCR	This study
	*aroA*_SF_rev	5’-GCCGACAATGTGCCGACGTTT-3’					This study
*xanQ*_1	*XanQ*_1_fw	5’-TCCCGCATCCGTTCAAATAC-3’	*xanQ*	84	84	PCR / qPCR	This study
	*XanQ*_1_rev	5’-CCAGCACGCTAAGCAGATAA-3’					This study
*xanQ*_2	*XanQ*_2_fw	5’-GCAGATGGTCTGGTTTCTGT-3’	*xanQ*	97	97	PCR / qPCR	This study
	*XanQ*_2_rev	5’-CGACGCCAGTCATCTGAATAA-3’					This study
*xanQ*_3	*XanQ*_3_fw	5’-CTGTCGGGTCATTACCCTTAAC-3’	*xanQ*	117	117	PCR / qPCR	[12]
	*XanQ*_3_rev	5’-AACCGAGGATAACCAGCATTAC-3’					[12]
*xanQ*_4	*XanQ*_4_fw	5’-TCCGCTGTCGGTTCATTAC-3’	*xanQ*	120	120	PCR / qPCR	This study
	*XanQ*_4_rev	5’-GCCGAGGATAACCAGCATTA-3’					This study
*xanQ*_SF	*XanQ*_SF_fw	5’-TCCCGCATCCGTTCAAATAC-3’	*xanQ*	498	498	Standard fragment PCR	This study
	*XanQ*_SF_rev	5’-CGCCAAGCCCTAAACCTAAA-3’					This study

SF = standard fragment

**Table 2:** List of probe sequences.

Name	Sequence	Source
Probe_*aroA*_1	5’-/56-FAM/CGGGTACTT/ZEN/ATTTGGTCGAAGGCGA/3IABkFQ/-3’	This study
Probe_*aroA*_2	5’-/56-FAM/CTTGATCCC/ZEN/AAATGCACGGCCAAA/3IABkFQ/-3’	This study
Probe_*aroA*_3	5’-/56-FAM/TCGGCTTCT/ZEN/TACTTTCTGGCAGCA/3IABkFQ/-3’	This study
Probe_*xanQ*_1	5’-/5HEX/CCTCCGCTG/ZEN/TCGGTTCATTACCAT/3IABkFQ/-3’	This study
Probe_*xanQ*_2	5’-/5HEX/TTCACGTTA/ZEN/TGTCGGGCGAACCAT/3IABkFQ/-3’	This study

HEX and FAM are fluorescent dyes, both 3IABkFQ (3’Iowa Black®FQ) and ZEN are quenchers

### PCR assay to determine the primer specificity

The specificity of the different PCR primer pairs was tested using a broad range of *E*. *coli* strains (mainly, but not exclusively, APEC), including 5 aromatic amino acid auxotrophic strains (*E*. *coli* strains harboring the WT *aroA* gene, but that are unable to synthesize aromatic amino acids), as well as other non-*Escherichia* strains (S1 Table). PCR was performed in a 20 μl total reaction mixture using 2 μl template DNA, 0.5 μM forward and 1 μM reverse primer in case of *aroA* primers; 0.5 μM in case of any other reverse primer and 2x biomix (Bioline, London, UK). Thermocycling was carried out in an Eppendorf Mastercycler Pro (Eppendorf, Hamburg, Germany) with the following parameters: 1 cycle at 94°C for 10 min followed by 30 cycles at 94°C for 45 s, 60°C for 30 s and 72°C for 45 s followed by 1 cycle at 72°C for 5 min. Agarose gel electrophoresis of the PCR products was performed on a 1.5% (w/v) agarose gel stained with MidoriGreen (Nippon Genetics, Tokyo, Japan) and visualized with UV-light. A 100-bp DNA ladder (GeneRulerTM, Thermo Scientific, Waltham, MA, USA) was used as a molecular weight marker.

### Determination of the efficiency and specificity of selected primers in a simplex qPCR reaction

To determine the efficiency of the developed primer pairs, a standard curve was generated for the mutated *aroA* gene and the *xanQ* gene using respectively the standard fragment primer pairs *aroA*_SF or *xanQ*_SF (Table 1) and DNA from the Δ*aroA* strain. The resulting PCR products were purified (MSB Spin PCRapace, Stratec Molecular, Berlin, Germany) and DNA concentration was determined spectrophotometrically (Nanodrop Technologies, Wilmington, DE, USA). The concentration of the linear dsDNA standard fragments was adjusted to 10^8^ - 10^0^ copies/μl, with tenfold dilution steps.

The specificity and efficiency of each of the primer pairs were assessed using a SYBRgreen qPCR assay. Each 12 μl qPCR reaction consisted of 2μl template DNA, 6 μl SensiMix™ SYBR® & Fluorescein Kit (Bioline), 0.5 μM forward primer and 0.5 μM reverse *xanQ* primer or 1 μM reverse *aroA* primer. Cycling was performed on a real-time PCR thermal cycler (Biorad, Hercules, CA, USA) and conditions were as follows: 95°C for 10 min, followed by 40 cycles of 95°C for 45 s and 62°C for 1 min. The fluorescent products were detected at the last step of each cycle. To confirm the specificity of the reaction, PCR products were subjected to melt analysis using a dissociation protocol comprising 95°C for 15 s, followed by 0.5°C incremental temperature ramping from 65°C to 90°C.

To develop a duplex qPCR reaction targeting both the total amount of *E*. *coli* as well as the amount of Δ*aroA* in a sample, the *xanQ* gene (total *E*. *coli*) and the *aroA* mutant gene (vaccine strain) need to be quantified using probes in different fluorescence channels. Therefore, primer pairs yielding good efficiency (90%-110% efficiency) in the SYBRgreen qPCR assay set-up were selected for further development in a probe-based qPCR assay. Each 12 μl of reaction mix consisted of 2 μl template DNA, 6μl 2X IQ Supermix (Biorad); 0.5 μM forward and 0.5 μM reverse *xanQ* primer or 1 μM reverse *aroA* primer and 0.2 μM probe. Cycling was carried out on a real-time PCR thermal cycler (Biorad) and conditions were: 95°C for 10 min, followed by 40 cycles of 95°C for 45 s and 62°C for 1 min with fluorescence detection of the products at the last step of each cycle.

For both the SYBRgreen qPCR assay and the probe-based qPCR assay, the efficiency of the assay was calculated by plotting the CT values of the standard fragment dilution series against the log standard fragment amount and determining the slope of the resulting standard curve. From the slope, efficiency was calculated using the following formula:

$$Efficiency\;(\%)=(10^{\left(\frac{-1}{slope}\right)}-1)*100$$

The limit of detection (LOD) of the assay was reported as the lowest template copy number yielding the correct melting temperature, whereas the limit of quantification (LOQ) was defined by the last valid Cq of the standard curve.

### Development of a probe-based duplex qPCR for detection of Δ*aroA* and WT *E*. *coli* in a single reaction

Two different primer-probe combinations targeting both the *aroA* mutant gene and the *xanQ* gene (total *E*. *coli*) were selected for further duplex probe-based qPCR development (Duplex qPCR A and B). The efficiency, LOQ and LOD of the qPCR reactions were determined in technical triplicates of a tenfold dilution series of both the *aroA* standard fragment and the *xanQ* standard fragment (10^8^–10^0^ copies/μl). Each 12 μl qPCR reaction contained: 2μl template DNA, 6 μl 2X IQ Supermix (Biorad); 0.5 μM forward, 0.5 μM reverse *xanQ* primer or 1 μM reverse *aroA* primer and 0.2 μM of each probe. Cycling was done on a real-time PCR thermal cycler (Biorad) with the following conditions: 95°C for 10 min, followed by 40 cycles of 95°C for 45 s and 62°C for 1 min, with fluorescence detection of the products at the last step of each cycle.

### Determination of possible effects of a faecal matrix on the final duplex probe-based qPCR

Since the duplex qPCR aims to discriminate Δ*aroA* from WT *E*. *coli* strains in faeces or intestinal contents from vaccinated chickens, the final selected primer-probe mixture (Duplex qPCR B) was further checked for any possible matrix effects. To assess the efficiency of the duplex qPCR assay, tenfold dilutions of the Δ*aroA* cultures were spiked into 100 mg of fresh chicken faeces, resulting in 10^9^ CFU/g-10^0^ CFU/g faeces, after which DNA was extracted with the CTAB method as described previously [14, 15]. The quality and the concentration of the DNA were examined spectrophotometrically (NanoDrop, Thermo Scientific, Merelbeke, Belgium), diluted to 50 ng/μl and stored at -20°C until further analysis.

To further assess the usability of the probe-based duplex qPCR to detect the Δ*aroA* strain, samples from both vaccinated and non-vaccinated birds were tested. Therefore, a total of 216 male Ross308 broilers were obtained from a local hatchery on day of hatch (Day1, 1-24h after hatching) and randomly allocated to 18 concrete floor pens (housed on wood shavings, 1m^2^, 12 birds per pen) separated by solid walls. On arrival, birds from 9 pens (vaccinated group) were vaccinated using the live attenuated Δ*aroA* vaccine strain (Poulvac® *E*. *coli*, dose: 10^8^ CFU/bird) in the drinking water. The nine other pens did not receive the vaccine (control birds). A commercial standard wheat/soy-based starter feed (Farm Mash 1, Versele Laga, Deinze, Belgium) and drinking water were provided *ad libitum*. At three days post-hatch, one bird per pen was euthanized to collect ileal content samples. All samples were stored at -20°C until further analysis. DNA was extracted and diluted to 50 ng/μl before performing the duplex qPCR as described above for the spiked faeces. Statistical analysis of the difference in total *E*. *coli* load between vaccinated and non-vaccinated birds was assessed using an unpaired t-test on the log-transformed data. The study followed the guidelines of the ethics committee of the Faculty of Veterinary Medicine, Ghent University, in accordance with the EU Directive 2010/63/EU.

## Results and discussion

### Development of a PCR technique to discriminate WT from Δ*aroA E*. *coli*

**Fig 2:**
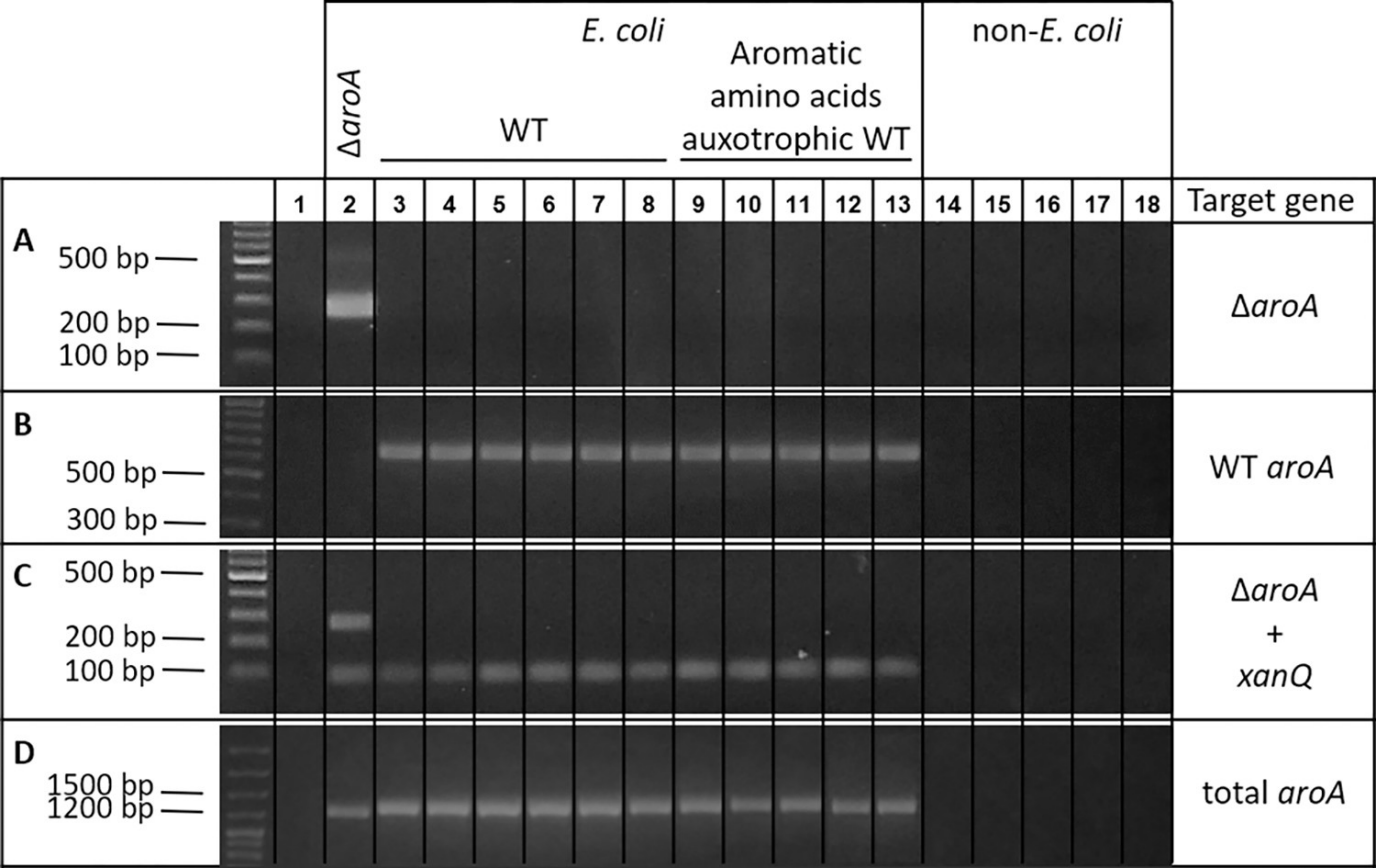
Discrimination of the Δ*aroA* vaccine strain from other *E*. *coli* strains and non-*E*. *coli* strains using PCR. The specificity of the primers targeting either the *E*. *coli* Δ*aroA* gene (A), the WT *E*. *coli aroA* gene (B), the previously published primers targeting both the WT and Δ*aroA* gene (total *aroA*) (D) or simultaneous detection of the Δ*aroA* and *xanQ* gene (C), was screened against a total of 132 *E*. *coli* strains and 7 non-*Escherichia* strains. (A) PCR specific for the Δ*aroA* gene. Amplification only occurred for the vaccine strain, while none of the commensal *E*. *coli* strains or non-*Escherichia* strains was amplified. The picture shows the results from primer pair *aroA*_3, which results in a band at 281bp when the *aroA* mutation is present. All other tested Δ*aroA* specific primers showed the same specificity. (B) The *aroA* primers targeting the WT gene (*aroA*_WT) result in a band at 660 bp in commensal *E*. *coli*, whereas no band occurred when the *aroA* mutation was present or in the non-*Escherichia* strains. (C) Duplex PCR with *aroA*_3 and *xanQ*_2 primers. The *E*. *coli*-specific *xanQ* primers result in a band at 97 bp, whereas the band at 281 bp indicates Δ*aroA*. Only for the Δ*aroA E*. *coli* strain, the two bands are present. (D) Primer pair *aroA*_LR (La Ragione et al., 2013, Table 1) is not specific for the Δ*aroA* mutation and results in an amplicon of 1236 bp for wild type *E*. *coli*, or 1161 bp for the Δ*aroA* vaccine strain. The pictures show a representative selection of the total number of strains used to assess the primer specificity. Samples: 1: blank control 2: *E*. *coli* O78:K80 Δ*aroA* (vaccine strain), 3: *E coli* O78:K80 WT, 4: *E*. *coli* Nissle 1917, 5: APEC_1 (chicken intestine), 6: APEC_2 (turkey intestine), 7: APEC019 (chicken intestine), 8: APEC023 (chicken intestine), 9: 220115–1 (chicken intestine, aromatic amino acid auxotrophic *E*. *coli*), 10: 220115–10 (chicken intestine, aromatic amino acid auxotrophic *E*. *coli*), 11: 06114–21 (chicken intestine, aromatic amino acid auxotrophic *E*. *coli*), 12: 7-35s (chicken intestine, aromatic amino acid auxotrophic *E*. *coli*), 13: 3-44s (chicken intestine, aromatic amino acid auxotrophic *E*. *coli*), 14: *Salmonella* Enteritidis, 15: *Salmonella* Typhimurium, 16: *Clostridium perfringens* NE18, 17: *Clostridium perfringens* CP20, 18: *Bacillus subtilis* DSM 29784. The complete list of tested strains can be found in S1 Table. Electrophoresis was performed on 1.5% agarose gel. GeneRulerTM 100 bp Plus DNA Ladder was used as a molecular weight marker.

At the start of this study, there was one known primer pair by La Ragione *et al*. (2013) that could differentiate between WT and the Δ*aroA E*. *coli*. This primer pair (*aroA*_LR, Table 1) targets the *aroA* gene, spanning the mutation, thereby resulting in a PCR amplicon of 1236 bp for the WT *E*. *coli* or 1161 bp for the Δ*aroA E*. *coli* [10]. Although this PCR has been used to discriminate WT from Δ*aroA* before [11], the small difference in amplicon length makes it hard to distinguish the vaccine strain from commensal *E*. *coli* strains (Fig 2D). Therefore, a novel PCR assay was developed to unambiguously differentiate the Δ*aroA* vaccine strain from commensal *E*. *coli* strains. The known mutation in the *aroA* gene was used to design the new primer pairs in the current study. Multiple primer sets for detecting Δ*aroA* and one pair to detect WT *E*. *coli* were generated (Table 1). The specificity of both the Δ*aroA* or WT PCR assays was assessed by screening different *E*. *coli* strains (mainly, but not exclusively, APEC) as well as other non-*Escherichia* strains (S1 Table). The *aroA*_WT primer set successfully amplified all WT *E*. *coli* strains, whereas no amplicon was observed for the Δ*aroA* vaccine strain, the non-*Escherichia* strains nor the water control (Fig 2B). All the *aroA* mutant primer sets in turn exclusively rendered an amplicon for Δ*aroA* and not for any other strain, also not the aromatic amino acid auxotrophic strains (particular *E*. *coli* strains that harbor a WT *aroA* gene, but are unable to synthesize aromatic amino acids), nor the water control (Fig 2A). Aromatic amino acid auxotrophic strains have previously been isolated from chicken intestinal content, and were shown to be phenotypically indistinguishable from the Δ*aroA* vaccine strain using standard plating methods [11]. Therefore, genetic methods, such as the *aroA* mutant primer sets developed in the current study, are needed to discriminate the Δ*aroA* vaccine strain from all commensal strains. Moreover, by performing a duplex PCR targeting both the *E*. *coli*-specific *xanQ* gene and the *aroA*-mutation, *E*. *coli* identification and detection of the vaccine strain can be performed in a single reaction (Fig 2C) [5, 12]. Moreover, inclusion of the *xanQ* gene in the duplex PCR serves as a control, as absence of a *xanQ* amplicon might indicate that the isolate is not an *E*. *coli* strain, or the DNA extraction or PCR reaction was not successful. As such, the PCR primers developed in our study give an easy, rapid and relatively cheap solution to discriminate the Δ*aroA* vaccine strain from other *E*. *coli* strains. Since the primers indicate whether the mutation is either present or not, the PCR results are unambiguously interpretable (Fig 2).

### Development of a probe-based duplex qPCR for quantitative detection of Δ*aroA* and WT *E*. *coli* in a single reaction

**Primer design and validation in single PCR.** To simultaneously detect the Δ*aroA* strain, as well as the total amount of *E*. *coli* in a sample, the *xanQ* gene was used as a target. This *xanQ* gene (also called *ygfO*) belongs to an evolutionarily conserved transporter family in *E*. *coli* [12], making it a functional target gene for an *E*. *coli* specific PCR. In total four different *xanQ* primer pairs were designed and tested for their specificity towards *E*. *coli* (*xanQ*_1 –*xanQ*_4; Table 1). All *xanQ* primer pairs were proven to be *E*. *coli* specific since no amplification occurred in non-*E*. *coli* DNA (Fig 2C).

**Determination of the efficiency of selected primers in a simplex qPCR reaction.** Different qPCRs were developed to quantify the amount of Δ*aroA* as well as the total amount of *E*. *coli* present in an individual sample. The four primer pairs for the *aroA* and the four pairs for the *xanQ* gene were first used in a SYBRGreen based qPCR to determine the efficiency of the qPCR reaction (Table 3). Therefore technical triplicates of a 10-fold dilution series of the standard fragments (10^8^−10^0^ copies/μl) generated with either *aroA*_SF or *xanQ*_SF were used (Table 1). The assays showing an efficiency between 90% and 110% in the SYBRgreen based qPCR were selected for further testing in a probe-based simplex set-up. This resulted in four *aroA* primer pairs and only two primer pairs for the *xanQ* gene. In none of the tested qPCRs amplification occurred in the negative controls (both water as well as non-specific DNA samples). The Cq values of the simplex probe-based qPCRs were comparable to those of the SYBRgreen assays (Table 3), indicating that the probe-based qPCR assay has the same working range as the SYBRgreen detection. Both *aroA* and *xanQ* probe-based qPCR assays had a comparable range of quantification, which was 10^2^−10^8^ copies/μl for the Δ*aroA* specific qPCR assays. The LOQ of the *xanQ* qPCR assay was slightly higher, with a quantification range of 10^2^-10^8^ copies/μl. For the *xanQ*_2 primers a higher LOQ (10^3^ copies/μl) was observed. The LOD was 10^4^ copies/μl. Since *xanQ* is *E*. *coli* specific and *E*. *coli* is a commensal, a higher LOQ has no impact on the usability of the qPCR set-up. The presence of a small amount (<10^4^ copies/ml) of *E*. *coli* in a certain sample will not be detected, but in the scope of this qPCR (detection of *E*. *coli* in faeces wherein *E*. *coli*–as a commensal- is always present in large amounts) it is negligible.

**Table 3:** Comparison of efficiency and limit of quantification in different PCR assays for all primer pairs. For each primer pair (Table 1) the efficiency, limit of quantification (LOQ) and limit of detection (LOD) were assessed using a 10-fold dilution series of a standard fragment (10^8^−10^0^ copies/μl).

	SybrGreen based qPCR					Simplex probe-based qPCR			
Primer pair	E (%)	LOQ (copies/μl)	LOD (copies/μl)	LOD (Cq)	Probe	E (%)	LOQ (copies/μl)	LOD (copies/μl)	LOD (Cq)
*aroA*_1	99.4	10^2^	10^3^	28	Probe_*aroA*_1	93.3	10^2^	10^3^	29
*aroA*_2	92.1	10^2^	10^3^	27	Probe_*aroA*_3	89.5	10^2^	10^3^	26
*aroA*_3	93.5	10^2^	10^3^	27	Probe_*aroA*_3	90.7	10^2^	10^3^	26
*aroA_*4	97.1	10^2^	10^3^	31	Probe_*aroA*_2	65	NA	NA	NA
*xanQ*_1	62.1	NA	NA	NA		ND	ND	ND	ND
*xanQ*_2	94.6	10^3^	10^4^	33	Probe_*xanQ_*1	108.1	10^3^	10^3^	29
*xanQ*_3	142	NA	NA	NA		ND	ND	ND	ND
*xanQ*_4	103.3	10^3^	10^4^	32	Probe_*xanQ_*2	92.4	10^2^	10^3^	32

ND = not determined, NA = not available, LOQ = limit of quantification, LOD = limit of detection, E(%) = efficiency. For each primer pair, a SYBR based qPCR with technical triplicates was run to determine efficiency, LOQ and LOD. A simplex probe-based qPCR with technical triplicates was run for those with efficiencies within the range of 90%-110%.

Only those primer pairs with an efficiency falling in the accepted range (90–110%) were withheld to perform a duplex probe-based qPCR, resulting in two *aroA* (*aroA*_1 and *aroA*_3) and two *xanQ* (*xanQ*_2 and *xanQ*_4) primer/probe combinations (Table 4).

**Table 4:** Comparison of efficiency, limit of quantification and limit of detection in the two different duplex probe-based qPCR set-ups. For each of the two primer/probe combinations the efficiency, limit of quantification and limit of detection were assessed using a 10-fold dilution series of a standard fragment (10^8^−10^0^ copies/μl). The choice for these specific combinations of primer pairs for both genes was based upon limited primer/probe interactions.

Duplex combination	primer pair	probe	E (%)	LOQ (copies/μl)	LOD	
					copies/μl	Cq
**Duplex qPCR A**	*aroA*_1	Probe_*aroA*_1	90,6	10^2^	10^3^	29
	*xanQ*_4	Probe_*xanQ*_2	88,6	10^3^	10^4^	30
**Duplex qPCR B**	*aroA*_3	Probe_*aroA*_2	90,9	10^2^	10^3^	26
	*xanQ*_2	Probe_*xanQ*_1	98,3	10^3^	10^4^	29

### Development of a probe-based duplex qPCR for simultaneous detection of Δ*aroA* and WT *E*. *coli*

A duplex qPCR was developed which allows the simultaneous quantification of both Δ*aroA* and commensal *E*. *coli* in the same sample, limiting possible technical errors or effects. At first, the two remaining *aroA* primer pairs (*aroA*_1/3) and the two remaining *xanQ* primer pairs (*xanQ*_2/4) were tested for possible primer/probe interactions by adding mismatches (e.g. *aroA*_1_fw with *xanQ*_2_rev) of primer/probes to a PCR reaction and see whether or not amplification occurred. Based upon these results two duplex primer/probe combinations were withheld, namely the combination of *aroA*_1 with *xanQ*_4 primers and *aroA*_3 and *xanQ*_2. The choice for those specific combinations of primer pairs for both genes was based upon the following criteria: (i) no amplification of the negative control; (ii) no non-specific interaction between the primers and probes of the two genes of interest. By performing qPCRs with altered primer/probe combinations, interactions between selected primers and probes for both genes were excluded. Cq values between simplex and duplex set-up differed less than 1 Cq, making it a successful duplex (Tables 3 and 4). The final choice to continue with the *aroA*_3 and *xanQ*_2 primer/probe combination was made based on the efficiency of the qPCR reaction. Because the *aroA* forward primer binds on the mutation whilst the *aroA* reverse primer binds on both the WT and Δ*aroA E*. *coli*, an increased need for reverse primer exists, which is solved by adding a double amount of reverse primer (1 μM as compared to 0.5 μM).

**Fig 3:**
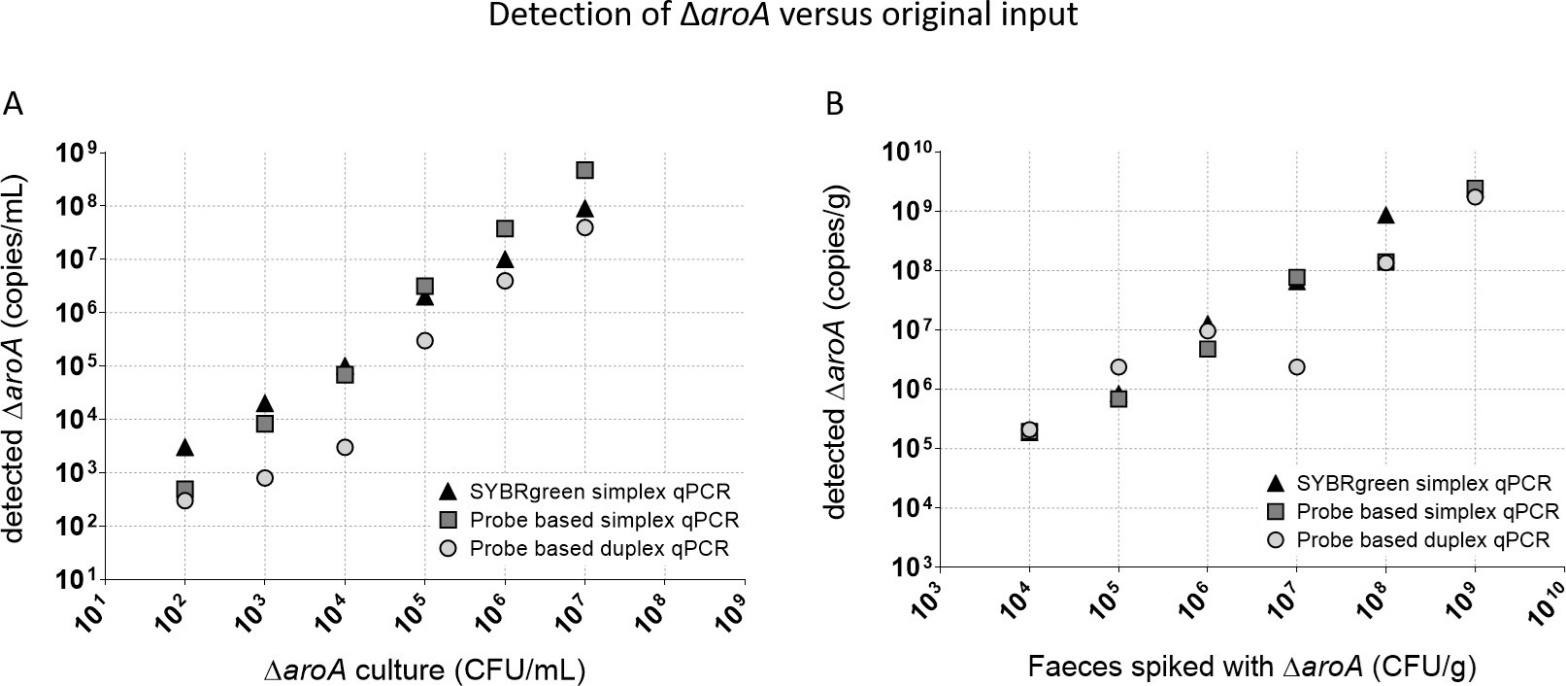
Amount of detected copies/ml Δ*aroA* vs original input. The amount of detected Δ*aroA* (copies/ml) for the three different qPCRs (the SYBRgreen simplex qPCR (black triangles), probe-based simplex qPCR (dark grey squares) and probe-based duplex qPCR (light grey circles) is depicted. (A) The input of the qPCR is genomic DNA originating from a 10-fold dilution series of an overnight culture from the Δ*aroA* strain. (B) The input for the qPCRs is DNA isolated from fresh chicken faeces spiked with a 10-fold dilution series of the Δ*aroA* strain.

**Specificity and analytical sensitivity of the duplex qPCR.** The analytical sensitivity was assessed by testing the LOD of the assay. Therefore, either spiked chicken faeces or 10-fold serial dilutions of genomic DNA from the Δ*aroA* strain was used. To quantify the copy numbers, a standard curve was generated from a 10-fold dilution series (10^8^−10^0^ copies/μl) of a standard fragment. The lowest template copy numbers yielding specific melting temperature in the SYBRGreen assays were considered as the detection limit of the assay (Table 3). To accurately detect the Δ*aroA* strain in a faecal sample, a minimum of 10^4^ CFU/g faeces is required for the duplex qPCR assay (Table 4 and Fig 3). In Fig 3, the amount of detected Δ*aroA* copies/ml versus the original input is depicted for both spiked faeces as well as for genomic DNA from the Δ*aroA* strain and for all three types of qPCR (SYBRgreen based, simplex and duplex probe-based). From this, it can be concluded that there is not a one on one relation between input and detection but that the detection still falls within reasonable deviation and this for all qPCR methods and both input methods.

**Fig 4:**
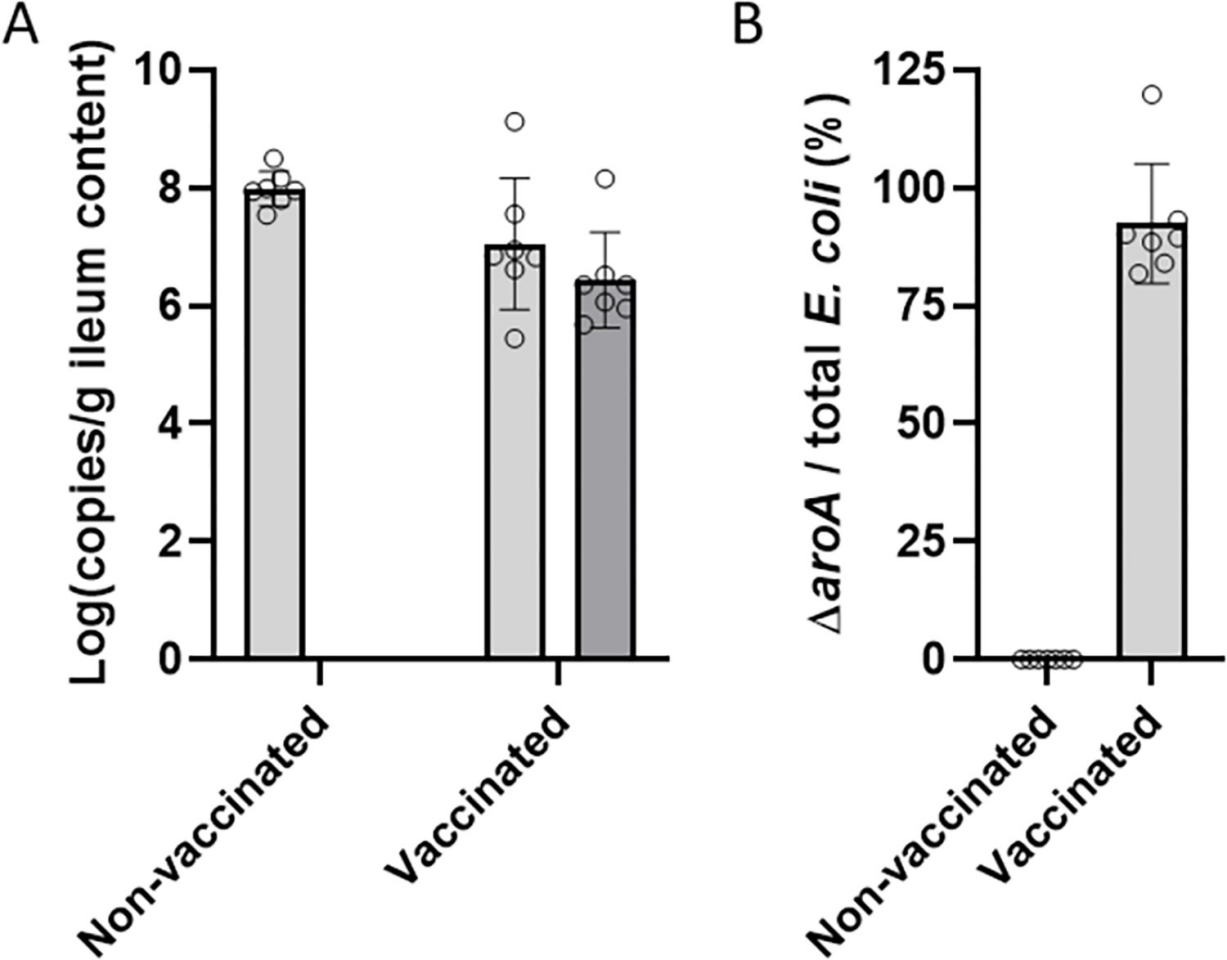
Detection of Δ*aroA* vaccine-strain and total *E*. *coli* load in ileal samples of vaccinated (n = 7) or non-vaccinated birds (n = 7). Birds from the vaccinated group received 10^8^ CFU Δ*aroA* vaccine per bird at day of hatch. At 3 days of age (2 days after vaccination), ileal content was collected. (A) Simultaneous detection of the vaccine strain (targeting the mutant *aroA* gene) (dark grey bars) and total *E*. *coli* load (targeting the *xanQ* gene) (light grey bars) was obtained using the duplex qPCR. Total *E*. *coli* load in the ileum of vaccinated birds tended to be lower than in non-vaccinated birds (p = 0.053). (B) 92.4% of the total *E*. *coli* load in the vaccinated birds was attributed to the Δ*aroA* vaccine strain, whereas in the non-vaccinated birds, no vaccine strain was detected.

To further confirm the usability of the developed duplex qPCR assay, the Δ*aroA* strain was quantified in ileal content samples from vaccinated birds. For this, birds were vaccinated with Δ*aroA* on day-of-hatch. On day 3 post-hatch, ileal content samples were collected from both vaccinated and non-vaccinated birds. Vaccination tended to reduce the total *E*. *coli* load in the ileum (*p* = 0.053). Moreover, no detection of vaccine strain occurred in non-vaccinated chickens, whilst the vaccine strain was detected in all birds in the vaccinated group (Fig 4), showing the specificity and the usability of the developed assay.

The sensitivity of the current duplex probe-based qPCR is adequate to prove the presence of Δ*aroA* in a particular sample in a quantifiable manner. The qPCR has a detection limit of 8.4*10^3^ copies/g faeces, which proved to be sufficient to detect the vaccine strain in intestinal content from vaccinated birds. When detection of lower amounts of vaccine shedding would be needed, it should be possible to perform an *E*. *coli* enrichment step prior to the PCR, after which solely the presence of the mutant strain can be proven but this no longer holds any quantification possibility.

## Conclusion

The PCR tools presented in our study are crucial in epidemiological investigations as well as for research purposes. They could also prove of value for monitoring the hygienic quality of meat or the presence of the vaccine strain on meat when optimized for this particular matrix. To develop a duplex qPCR for the simultaneous quantification of the Δ*aroA* vaccine and total *E*. *coli* load in a specific sample, this study made use of: (i) the genetic *aroA* mutation inserted by allelic exchange [10] and (ii) *E*. *coli* specific primers targeting the *xanQ* gene [12]. The qPCR has a detection limit for Δ*aroA* of 8.4 *10^3^ copies/g faeces as was determined by spiking fresh chicken faeces with a 10-fold dilution series of Δ*aroA*, and it is specific for this vaccine strain as it did not pick up any signal in control intestinal samples from non-vaccinated chickens. The set-up was optimized for DNA samples from chicken intestinal content.

This fast, easy-to-use and relatively cheap tool for discriminating the Poulvac® *E*. *coli* vaccine strain from other *E*. *coli* strains provides specific identification and quantification of the *aroA* mutant, making it ideal for screening bacterial populations retrieved from chicken faeces or intestinal content. The duplex qPCR is helpful in monitoring colonization and shedding of the vaccine strain.

## Supporting information

S1 Table List of all used bacterial strains in this study. (XLSX)

S1 File Raw gel images used to construct Fig 2. (DOCX)
