# Supplementary material for: Development of a duplex qPCR for the differentiation of a live attenuated Escherichia coli aroA mutant vaccine strain from field isolates in chickens
Source: PLoS One. 2022 Dec 19;17(12):e0278949. doi: 10.1371/journal.pone.0278949 (PMC9762556; doi:10.1371/journal.pone.0278949)

Raw gel image 1.

Top row: samples from an unrelated project

Second row: Fig 2B (WT *aroA* gene detected using primer pair *aroA*_WT)

Third row: Fig 2D (detection of both the WT *aroA* and mutant *aroA* gene using primer pair *aroA*_LR)


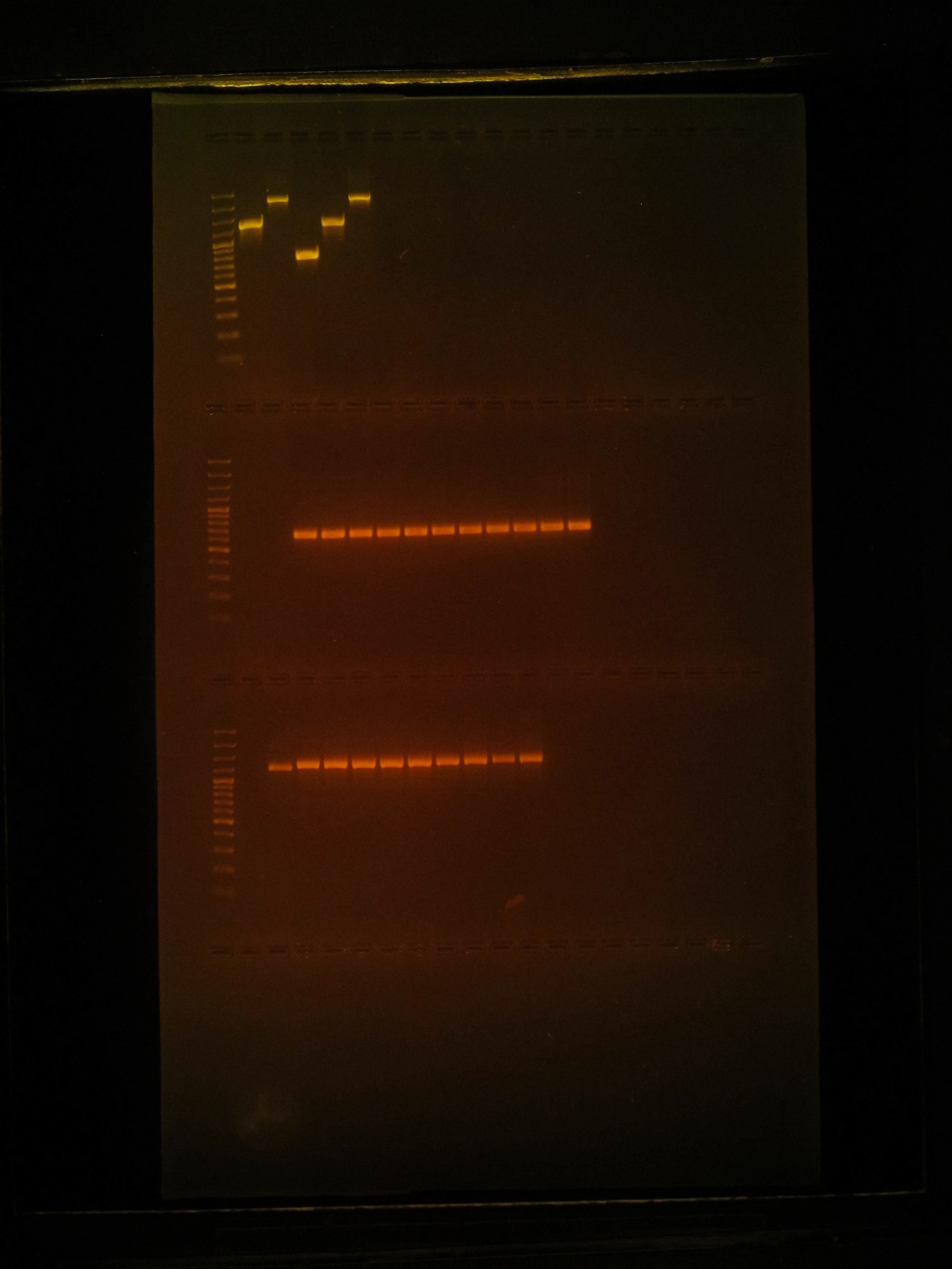


Raw gel image 2.

Top row: Fig 2A (mutant *aroA* gene specifc primers)

Third row: Fig 2C (duplex PCR using primer pairs *aroA*_3 and *xanQ*_2)

Second and bottom row: amplicons from an unrelated project


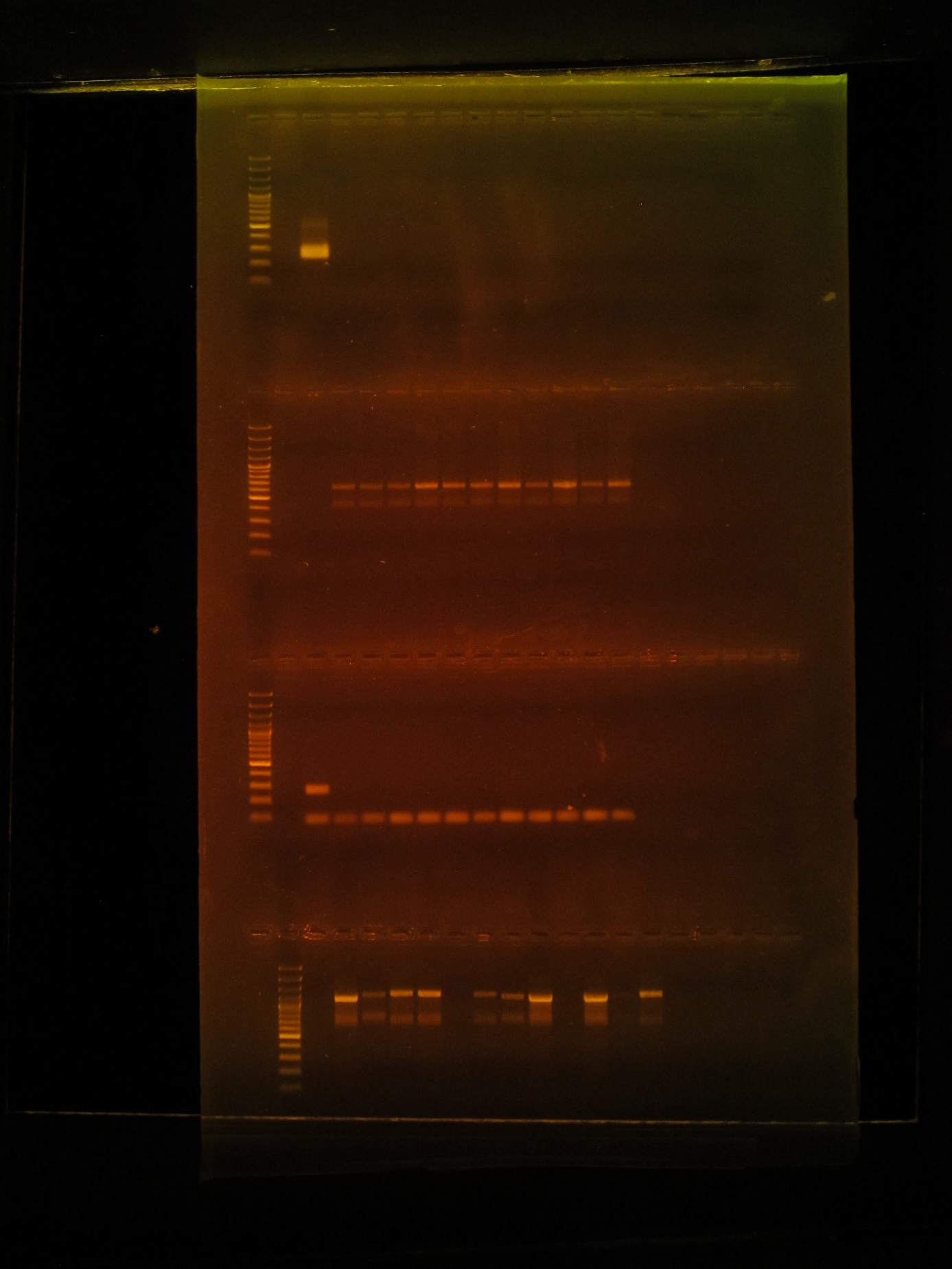

Supplement: S1 File — (DOCX) [file pone.0278949.s002.docx]
